# Heredity in Monkeys

**Published:** 1892-03

**Authors:** 


### HEREDITY IN MONKEYS.

An American of ample means has recently declared his intention, privately, to devote $100,000 to a very orginal purpose.

The idea is that no satisfactory opportunity has ever been afforded for the development of the intellect of the brute. Intelligence, like bodily qualities, is susceptible of improvement through breeding, as every one knows who has thought about the evolution of the dog from the wolf by artificial selection. This rich man proposes monkeys or apes shall be taken as subjects for experiment, simply because man understands those animals better than he does others.

Let fifty of them, half males and half females, be placed in a paddock, suitably provided with separate quarters for the sexes. Then have them bred, pair by pair, as shall be directed by those who superintend. Some of them will develop certain abilities more conspicuously than others. For example, certain individuals will exhibit a superior understanding of the commands addressed to them or will show a greater dexterity in the handling of objects. Those which appear stupid are to be expelled from the colony, their places being filled by fresh recruits. When a male and a female are found who exhibit the same sort of aptitude in any direction they are to be mated. This process, carried on for generations, would necessarily result in the development of superior characters, until finally, after the lapse of twenty-five years, perhaps, there would almost certainly be had apes or monkeys far higher in the scale of reason than any known up to the present time.

These putative cousins of the human race have already exhibited a mental and even mechanical capacity sufficient to give ground for great hopes of possible development in point of intellect. Chimpanzees have been taught to bring things to the table, and the big Langur baboon of India is commonly used in that country to-day as a servant for working the pukah fans, with which flies are kept away from dinner tables. Explorer Stanley has given an account of apes which carry torches at night.

This is believed by scientists to be an absurdity, because all the anthropoid and simian tribes are too afraid of fire to render such a thing possible. It is a fact well known that gorillas, while they will gather about a deserted camp fire for the sake of warmth, will never think of keeping the embers alight by adding fuel. Nevertheless every book on natural history relates many an instance illustrative of these creatures' thinking powers, and there is no question that it could be greatly improved by the process of judicious breeding. Even a pig can be taught to count up to ten.

